# Effect of indwelling depth of peripheral intravenous catheters on thrombophlebitis

**DOI:** 10.1097/MD.0000000000034427

**Published:** 2023-07-21

**Authors:** Chenghong He, Yujing Shi, Xu Jia, Xihui Wu, Qian Xing, Liang Liang, Mengyang Ju, Xiaoke Di, Yin Xia, Xiaojiao Chen, Jun Shen

**Affiliations:** a Department of Oncology, Jurong People’s Hospital, Zhenjiang, Jiangsu, China; b Department of Oncology, Jurong Hospital Affiliated to Jiangsu University, Zhenjiang, Jiangsu, China; c Department of Radiation Oncology, Osaka University Graduate School of Medicine, Suita, Osaka, Japan; d Department of Radiation Oncology, First Affiliated Hospital of Nanjing Medical University, Nanjing, Jiangsu, China.

**Keywords:** indwelling depth, intravenous catheters, thrombophlebitis, tumor

## Abstract

To clarify the effect of catheter indwelling depth on the occurrence of thrombophlebitis, a total of 339 hospitalized patients were randomly enrolled and divided by the catheter indwelling depth into 2 groups. Then the effect of indwelling depth on thrombophlebitis was analyzed, and the independent influence factors on the occurrence of thrombophlebitis were clarified. There were 49 cases of thrombophlebitis, including 8 tumor-bearing patients and 41 patients with lung infection. Thirteen of the 135 patients with indwelling depth of 1 cm, and 36 of the 204 patients with indwelling depth of 1.9 cm suffered thrombophlebitis. The relationship between incidence rate of thrombophlebitis and clinicopathological parameters was analyzed. It was found the incidence of thrombophlebitis was significantly correlated with males (X^2^ = 5.77), lung infection (X^2^ = 7.79), and indwelling depth of 1.9 cm (X^2^ = 4.223). Multifactor analysis of variance showed the significant independent risk factors of thrombophlebitis were male [hazard ratio (HR) 3.12 (1.39–6.98)], and lung infection (HR 0.22 [0.06–0.69]), and the indwelling depth of 1.9 cm affected the occurrence of thrombophlebitis (HR 0.79 [0.42 –3.09]) but was not an independent risk factor. In our treatment center, while appropriate fixation was ensured, the catheter indwelling depth shall be as short as possible, so as to reduce the occurrence of thrombophlebitis. For patients with lung infection, nursing at the intubation site shall be strengthened, so as to decrease thrombophlebitis.

## 1. Introduction

In modern medical practice, peripheral intravenous catheters (PIVC) is widely applied in in-hospital and outpatient services.^[1]^ However, the complications of PIVC (e.g., infiltration, exosmosis, blockage, dislocation, phlebitis) subject 69% of patients to premature failure, which calls for insertion of new facilities and thus delays treatment and increases costs.^[2]^ In fact, thrombophlebitis is a common and severe complication of PIVC.^[3–5]^ Thrombophlebitis refers to 2 or more of the following symptoms or signs at the catheter site or near adjacent veins: pains, tenderness, erythema, swelling, suppuration, and touchable vein swelling.^[6–8]^

The mechanism of phlebitis is believed to be a result from multifactor effects on venous walls, including chemical irritation, bacterial pollution, and machinery traumas. Chemical irritation often occurs after the infusion of vesicular agent or irritating solutions, or solutions related to potential endothelial injuries during peripheral catheter administration. Bacterial phlebitis is usually induced by extraluminal pollution (from skin floras). Mechanical damages often occur after inappropriate fixation of catheters, or insertion of a large catheter into venules, and the subsequent friction of the catheter on vein walls as well as endothelial injury.^[9]^ Besides, the occurrence of thrombophlebitis is also affected by the personal factors of patients, including history of thrombus, trauma,^[6]^ immune function deficiency,^[10]^ complications,^[11]^ diabetes,^[12]^ malignancy,^[13]^ and high hemoglobin level.^[14]^ However, the effect of the indwelling depth of PIVC on the occurrence of thrombophlebitis has been rarely reported. Herein, the clinical cases in the therapy center of our hospital were collected to further analyze how the indwelling depth of PIVC will affect the occurrence and development of thrombophlebitis and thus to further optimize the clinical practice of our hospital.

## 2. Materials and methods

### 2.1. Basic information

A total of 376 in-hospital patients treated in the tumor department or the respiratory department of our hospital between March and August 2022 were randomly enrolled. The inclusion criteria were: Zubrod ECOG WHO performance status score = 0 to 2; Complete medical record; Age = 18 to 85 years; No evident hemorrhagic complication. The exclusion criteria were: Zubrod ECOG WHO performance status score > 2; age > 85 years; Incomplete clinical data; Activity-caused bleeding; Critical infection or infection-induced shock. The chemotherapeutic drugs for all tumor-bearing patients were made via peripherally inserted central catheters. Basic information including history of complications, history of thrombus, platelet count before intubation, D-dimer, and partial prothrombin time was collected.

### 2.2. Methods

**Figure 1.**
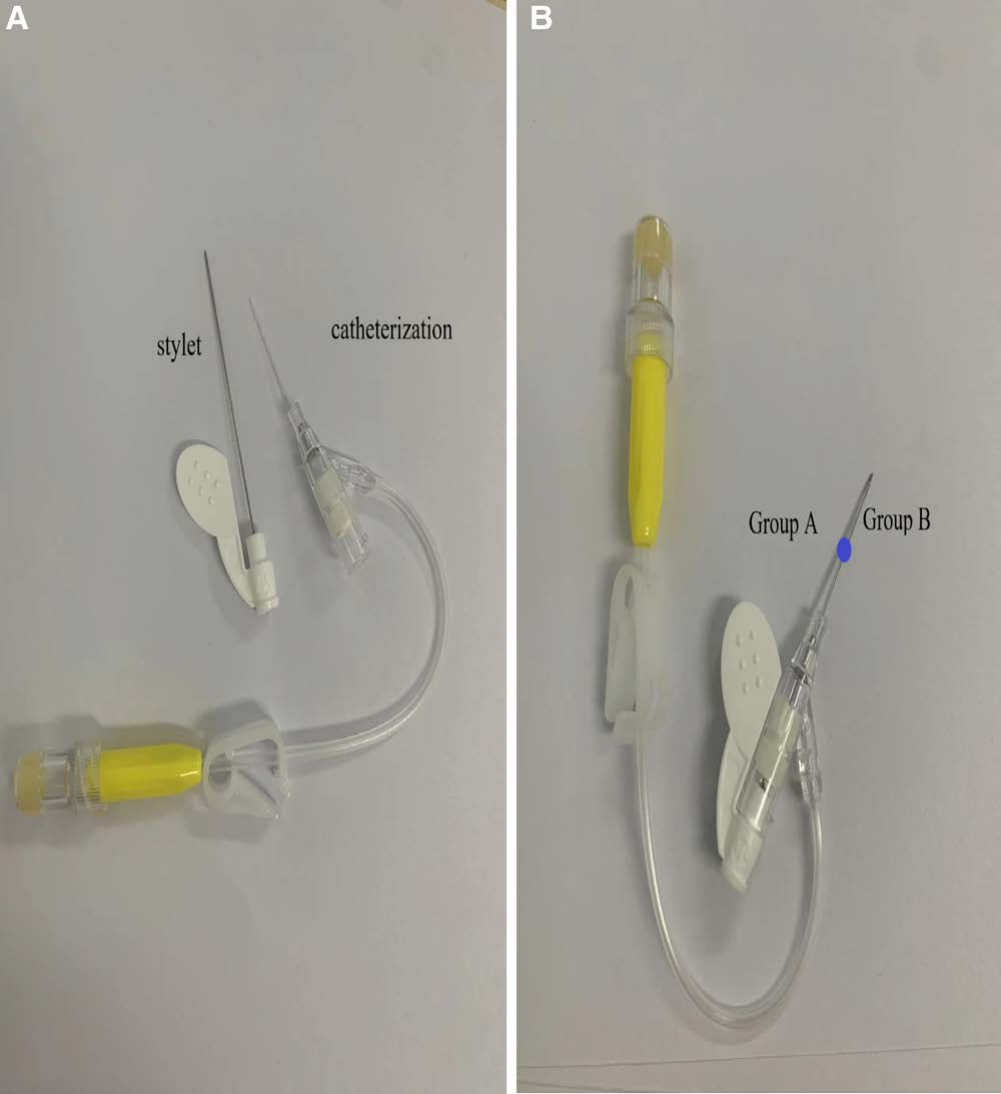
(A). Clinical indwelling catheter, (B) grouping by catheter indwelling depth: whole length of anterior indwelling catheter in Group A, and whole length from central blue point to anterior part in group B.

**Figure 2.**
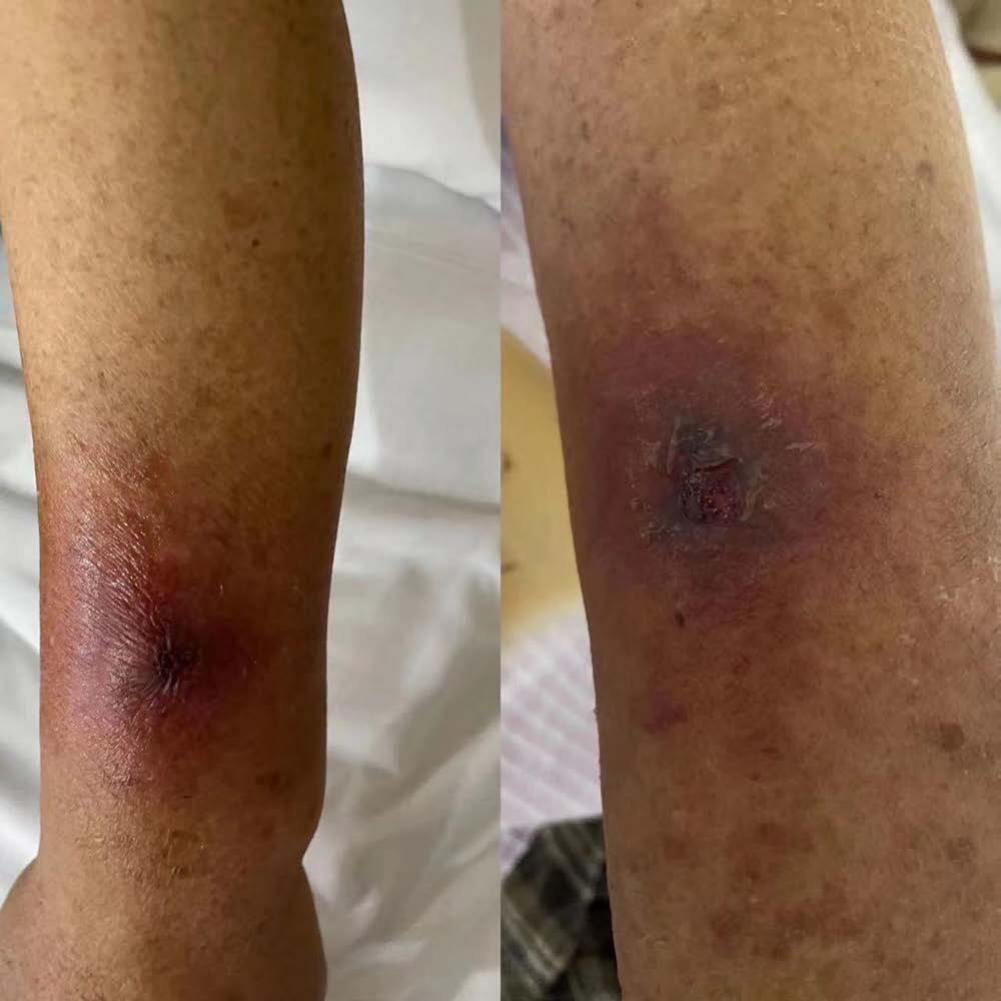
Clinical manifestations of thrombophlebitis.

The patients were divided by the indwelling depth into groups A and B, in which the intravascular depth of PIVC was 1.9 and 1 cm respectively. All patients were assessed by directors and nurse practitioner of our hospital to be qualified for enrollment. Then their vascular conditions were evaluated before intravenous cathetering. The blood vessel to be chosen was forearm or upper arm veins, which avoided the veins in any paralyzed limb or lower limb. The vein indwelling needle was the 24 Gauge (Ga.) 19 mm catheter (Suzhou Yuwell Medical Technology Co., Ltd.). Local skins were all complete without injury or ulceration and were not applied with antibacterial grease. Prior to aspiration, the aspiration site was disinfected (8 × 8 cm2) and left to dry. Then the core of the indwelling needle was loosened at left and right sides, and the needle was inserted at an angle of 15° to 30°. When blood appeared, the needle was lowered to an angle of 5° to 15° and further moved 0.2 cm. Then the needle core was returned by 0.5 to 1 cm. In group A, each PIVC catheter was totally inserted to blood vessels (1.9 cm). In group B, each PIVC catheter was inserted by 1 cm to blood vessels (Fig. 1). Then each catheter was fixed with 3M transparent paste (6 × 7 cm^2^). When the patient developed discomfort at the insertion site or when the dressing came loose, the dressing was removed, the site was examined, then disinfected with 0.5% iodophor in preparation for a dressing change, and then resecured. When the symptoms stated above occurred, diagnosis was made as per thrombophlebitis assessment scale^[4]^ (Fig. 2). To clarify the effect of PIVC indwelling depth on the occurrence of thrombophlebitis, we probed into other influence factors on thrombophlebitis through stratified analysis. This study was approved by the Ethics Committee of Jurong People’s Hospital (JRSRMYY-2022-006). All patients signed informed consent.

## 3. Statistical methods

Statistical analysis was conducted on SPSS 23.0 (IBM SPSS Statistics, Armonk, NY: IBM Corp) and Graphpad (GraphPad Software, Boston, MA). The effect of catheter indwelling depth on the occurrence of thrombophlebitis was analyzed via χ^2^ test. The influence factors on thrombophlebitis were explored via single-factor logistic regression analysis. All factors were sent to multifactor Cox regression analysis. The hazard ratio (HR) of each factor was computed. The odds ratio, HR, and corresponding confidence interval at 95% level were determined. All tests were 2-tailed significance tests at α = 0.05, unless otherwise noted. *P* < .05 indicates significance.

## 4. Results

### 4.1. Baseline information

Of the 376 patients, 8 cases with emerging gastrointestinal bleeding or hemoptysis, 22 cases with intubation time < 24 hours (7 cases in group A, 15 cases in group B), 4 cases with tube fall-off (3 cases in group A, 1 case in group B), and 3 cases who withdrew spontaneously (1 case in group A, 2 cases in group B) were excluded. Finally, 339 patients were enrolled, including 204 cases in group A and 135 cases in group B. The basic information of clinicopathological factors from the 2 groups was listed in Table 1.

**Table 1:** Patients information.

Characteristics	
Gender	
Male	224
Female	115
Age (yr)	
Range	19-85
Average	68.6
ZPS score	
0–1	309
2	30
Disease type	
Tumor	118
Lung infection	221
Diabetes	
Yes	65
No	274
Thrombus	
Yes	3
No	336
Platelet count	
≤300	303
>300	36
APTT	
≤44s	335
>44s	4
D-dimer	
≤0.5	151
>0.5	188
Anticoagulant	
Yes	2
No	337
Indwelling depth	
1cm	135
1.9 cm	204
Indwelling site	
Forearm	277
Other	62
Indwelling time	
≤3 d	110
>3 d	229
Block pipe	
Yes	12
No	327
Effusion	
Yes	14
No	325
Errhysis	
Yes	26
No	313
Nutrition-score (NRS2002)	
0–1	232
2–3	107

ZPS = Zubrod ECOG WHO performance status.

Table 1 Basic information of clinicopathological factors from groups A and B.

### 4.2. Relationship between thrombophlebitis and clinicopathological factors

There were 49 cases of thrombophlebitis, including 8 tumor-bearing patients and 41 non-tumor patients. Thirty 6 of the 204 patients in group A, and 13 of the 135 patients in group B suffered thrombophlebitis. Thrombophlebitis occurred on the 2nd day in 5 patients (10.2%) and on the 3rd day in 20 patients (40%). The relationship between incidence rate of thrombophlebitis and clinicopathological parameters was analyzed. It was found the incidence of thrombophlebitis was significantly correlated with males (X^2^ = 5.77 *P* = .016), and non-tumor disease (X^2^ = 7.79 *P* = .006). The relationship was significant in group A (X^2^ = 4.223 *P* = .04). Age, diabetes, history of thrombus, platelet count, prothrombin time, D-dimer, catheter indwelling time, or catheter indwelling position was not significantly correlated with the occurrence of thrombophlebitis (all *P* > .05) (Table 2).

**Table 2:** Relationship between occurrence of thrombophlebitis and clinicopathologic factors.

Characteristics	Yes (49)	No (290)	*P* value
Gender			.016*
Male Female	40 9	184 106	
Age (yr)			.49*
>65 ≤65	33 16	210 80	
ZPS			.37*
0–1 2	42 7	267 23	
Tumour			.006*
Yes No	8 41	110 180	
Diabetes			.66*
Yes No	8 41	53 237	
Platelet count			.804†
>300 ≤300	3 46	33 257	
Prothrombin time			.47†
>44s ≤44s	1 48	3 287	
D-dimer			.44*
>0.5 ≤0.5	30 19	157 133	
Nutrition score (NRS2002)			.37*
0–1 2–3	28 21	194 96	
Indwelling depth			.04*
1 cm 1.9 cm	13 36	122 168	
Indwelling time			.052*
>3 d ≤3 d	35 14	194 96	
Indwelling site			.99*
Forearm Others	40 9	237 53	

* *Chi square test.*

† *Fisher exact test.*

Table 2 Relationship between thrombophlebitis and clinicopathological factors.

### 4.3. Independent influence factors of thrombophlebitis

The relationship between occurrence of thrombophlebitis and clinicopathological parameters was analyzed. It was found the incidence of thrombophlebitis was significantly correlated with gender, non-tumor disease, and indwelling depth (all *P* < .05). Then the above 3 factors were sent to multifactor analysis of variance. Results showed the significant independent risk factors of thrombophlebitis were male (HR 3.12 [1.39–6.98], *P* = .006), and non-tumor disease (HR 0.22 [0.06–0.69], *P* = .01). The indwelling depth affected the occurrence of thrombophlebitis (HR 0.79 [0.42–3.09], *P* = .79), but was not an independent risk factor (Table 3).

**Table 3:** The influencing factors of thrombophlebitis were clarified by univariate and multivariate analysis of variance.

Characteristics	Univariable		Multivariable	
	HR with 95% CI	*P* value	HR with 95% CI	*P* value
Age (yr) ≤65 >65	1.3 (0.68–2.48)	.44		
Gender Male Female	0.4 (0.19–0.86)	.02	3.1 (1.39–6.98)	.006
Tumour Yes No	3.11 (1.35–7.18)	.008	0.22 (0.06–0.69)	.01
ZPS 0–1 2	0.73 (0.48–1.11)	.15		
Diabetes Yes No	0.83 (0.37–1.88)	.66		
Platelet count >300 ≤300	0.89 (0.35 –2.25)	.804		
Prothrombin time >44 ≤44	1.99 (0.20–19.56)	.55		
D-Dimer >0.5 ≤0.5	1.32 (0.71–2.45)	.38		
Indwelling depth 1 cm 1.9 cm	0.5 (0.25–0.98)	.04	1.15 (0.42–3.09)	.79
Indwelling time >3 d ≤3 d	1.26 (0.65–2.45)	.50		
Indwelling site Forearm Others	0.99 (0.46–2.17)	.99		
Effusion Yes No	2.49 (0.75–8.28)	.14		
Errhysis Yes No	1.08 (0.36–3.29)	.89		
Nutrition score 0–1 2–3	0.64 (0.35–1.2)	.16		

CI = confidence interval, HR = hazard ratio.

Table 3 Independent influence factors of thrombophlebitis.

## 5. Discussion

Upper extremity superficial vein thrombophlebitis (UESVT) is relatively common. The imaging criterion of UESVT is that the upper extremity superfine-fiber venous segment is incompressible on ultrasonic images.^[15]^ Its pathogenesis is considered to be thrombosis due to venous wall inflammation. Though UESVT is regarded as a negligible complication, it may cause severe discomfort and calls for repeated intubation. Recurrence of UESVT can complicate vein insertion and calls for the placement of central venous catheter, which will delay parenteral administration and prolong in-hospital time. Besides, severe complication may occasionally occur, such as suppurative phlebitis and sepsis.^[15]^ Researchers have studied the effects of intubation material, catheter indwelling time, complications, and local skin inflammation on thrombophlebitis, but have not reported the effect of indwelling depth. Reportedly, the incidence rate of thrombophlebitis is 20% to 80%.^[16]^ Maki and Ringer compared the effects of 2 intubation materials on thrombophlebitis and found the total incidence rate of thrombophlebitis among 714 patients was 42%, including 30% and 45% of cases occurring on the 2nd day and the 3rd day respectively.^[6]^ Nicholas Mielke used ultrasound into diagnosis of thrombophlebitis and found the incidence rate of thrombophlebitis among 62 included patients was 87.10%.^[17]^ Among the 339 patients in the present study, the incidence rate of thrombophlebitis is 14.5%, which is inconsistent with previous studies and is directly related to the normalized nursing and the optimized consumable quality of our hospital. Moreover, our nursing is timely and active; after use of high-concentration or irritant drugs, the indwelling needles and tubes are rinsed with normal saline; activities of the body at the intubation side are well guided to promote blood circulation. All these measures are inseparable and increase the incidence rate above.

The 339 included patients involved 118 tumor-bearing patients and 221 non-tumor patients. The occurrence rate of thrombophlebitis among the tumor-bearing patients was 6.7%, which was significantly lower than that among the non-tumor patients (18.5%). However, this result is inconsistent with another study that the incidence rate of thrombophlebitis is higher among tumor-bearing patients.^[18]^ The first reason for this inconsistency is that the intravenous chemotherapy of all tumor-bearing patients in our study was conducted via peripherally inserted central catheters, and no infusion of chemotherapeutic drugs was done in PIVC, which together led to the relatively low incidence rate of phlebitis in our study. Secondly, the non-tumor patients included our study were patients with respiratory infection, so the infusion rate of antibiotics and other irritant drugs was significantly higher than among the tumor-bearing patients. Hence, the incidence rate of phlebitis was higher than the tumor-bearing patients. This result is consistent with another study that the incidence rate of thrombophlebitis was higher among patients with infectious diseases or infused with antibiotics.^[16]^ Diabetes is a high-risk factor of thrombophlebitis.^[13,19]^ Saji et al^[20]^ found the incidence rate of thrombophlebitis among diabetic patients was 40% higher than among nondiabetic patients. However, Monreal et al found diabetes was not an independent risk factor of thrombophlebitis, which is consistent with our findings. Among the 339 included patients, only 8 of the 61 diabetic patients suffered thrombophlebitis. Moreover, 1-factor analysis of variance showed diabetes was not an independent risk factor of thrombophlebitis (HR 0.83 [0.37–1.88], *P* = .66). As for the intubation position, reportedly, the risk of thrombophlebitis at forearm or elbow anterior nest was higher than wrists or the back of hand,^[16]^ which is similar to our findings. Furthermore, the incidence rate of thrombophlebitis among non-tumor patients was significantly higher than among tumor-bearing patients. As for the reasons, the average indwelling time of the tumor-bearing patients was 3.35 days, and was 4.54 days among the non-tumor patients.

Collin J et al^[21]^ found the peripheral vein tube length was also related to the occurrence of peripheral intravenous thrombophlebitis. On this basis, the 339 patients were divided by the indwelling depth into 2 groups. It was found the indwelling depth affected the occurrence of thrombophlebitis but was not an independent risk factor. The incidence rate of thrombophlebitis among the patients at the indwelling depth of 1.9 cm was higher (36/168), which may be ascribed to the altered blood flow direction, slower blood flow, and thrombosis due to the deeper intubation site. Dar Weiss used short peripheral catheters into the veins of a pig model, and proved that shorter indwelling catheters significantly decreased catheter-related phlebitis in vivo,^[22]^ which is consistent with our findings.

This study still has some limitations. First, this is a single-center study, which may cause bias, so multi-center collaborative research is needed to validate our findings. Second, the sample size is relatively small, so our findings shall be further validated. Finally, the included patients suffer tumors and lung infection, so patients of other diseases are needed to confirm our results.

## 6. Conclusion

Peripheral vein thrombophlebitis is a common complication during the use of peripheral vein catheters, and its incidence rate is 2.5% to 80%. As the specific risk factors of catheters are increasingly concerned, better description of biological factors will improve our understanding about the pathogenesis of peripheral vein thrombophlebitis and can help to establish better management strategies. In our single-center study, shorter length catheters were associated with a lower occurrence of thrombophlebitis.

## Acknowledgements

This study was funded by the Research project of clinical medical science and technology development fund of Jiangsu University (No: JLY2021097); Court-level Natural Science Foundation Project of Jurong People’s Hospital (No: JY20221001), National Natural Science Foundation of China (82003228), Jiangsu Science and technology project (grant BK20201080), Zhenjiang Science Technology Guidance Project (FZ2020016) and Jurong City Social Development Project (ZA42112).

## Author contributions

**Conceptualization:** Jun Shen, Yujing Shi, Yin Xia.

**Data curation:** Jun Shen, Xu Jia, Mengyang Ju, Yin Xia.

**Formal analysis:** Chenghong He, Jun Shen, Mengyang Ju, Xiaoke Di.

**Funding acquisition:** Jun Shen.

**Investigation:** Yujing Shi, Xihui Wu, Xiaoke Di.

**Methodology:** Chenghong He, Yujing Shi, Qian Xing, Mengyang Ju.

**Project administration:** Chenghong He, Mengyang Ju, Xiaojiao Chen.

**Resources:** Xihui Wu, Xiaoke Di.

**Software:** Chenghong He, Yujing Shi, Xiaoke Di.

**Supervision:** Jun Shen, Liang Liang.

**Validation:** Qian Xing.

**Visualization:** Liang Liang.

**Writing – original draft:** Chenghong He, Yujing Shi.

**Writing – review & editing:** Chenghong He, Yujing Shi, Liang Liang.
